# Government preparedness and response towards COVID-19 outbreak in Nigeria: A retrospective analysis of the last 6 months

**DOI:** 10.7189/jogh.10.020382

**Published:** 2020-12

**Authors:** Oluwafolajimi A Adesanya

**Affiliations:** Institute for Advanced Medical Research and Training (IAMRAT), College of Medicine, University of Ibadan, Ibadan, Nigeria

In December 2019, a cluster of atypical cases of pneumoniae were reported in individuals who had come in contact with the Huanan Seafood Market in Wuhan, China. The causative agent was soon identified to be SARS-CoV-2 – a novel member of the β-coronaviridae family [1]. By the end of January 2020, the World Health Organisation (WHO) had declared the outbreak a Public Health Emergency of International Concern (PHEIC), and by March 11, 2020, a full-blown pandemic [2]. On February 27, 2020, Nigeria recorded its first case of COVID-19 in an Italian man who had gained entry into the country over 48hours earlier and travelled inter-state before developing symptoms and eventually deciding to self-isolate [3]. This incident marked the onset of the COVID-19 outbreak in Nigeria, resulting in 46 577 confirmed cases and 945 deaths by August 10, 2020 [4]. This viewpoint evaluates some of the preparedness/response strategies deployed by the Nigerian Government to flatten the curve (**Figure 1**), by highlighting the series of events that have played out before and after the arrival of patient zero.

**Figure 1:**
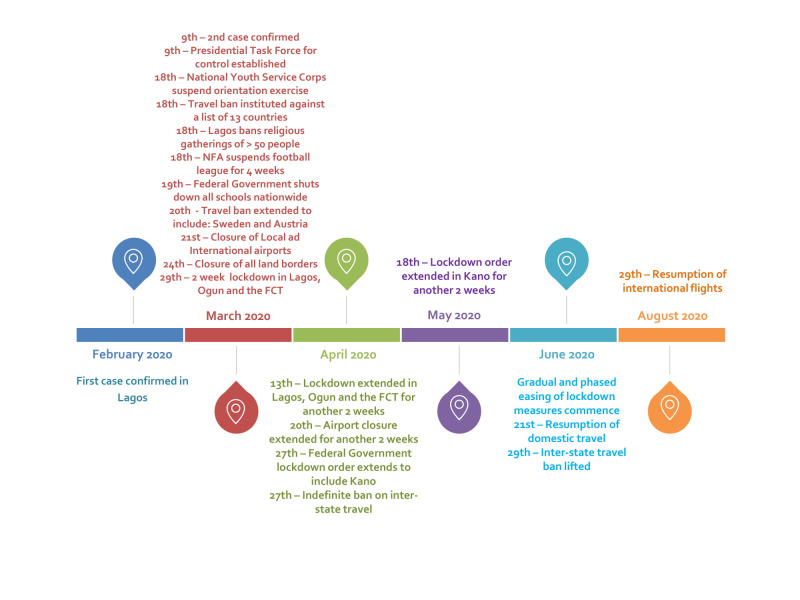
Timeline showing the COVID-19 response strategies employed by the Nigerian Government between February and August 2020.

## BEFORE THE INDEX CASE

Prior to the arrival of patient zero, the Federal Government through its disease outbreak response agency – the Nigeria Centre for Disease Control (NCDC), had taken certain measures to prevent the entry of the virus into the country or ensure its rapid detection on arrival. Some of those measures included: increased surveillance at ports of entry through temperature checks, travel history documentation and collection of contact details of passengers arriving from COVID-19 hotspots; activation of the National Incident Coordination Centre (ICC) for coordination of preparedness/response efforts and the development of the Surveillance and Outbreak Response Management System (SORMAS) and the Mobile Strengthening Epidemic Response System (mSers), which were instruments of the ICC deployed for case-based reporting and aggregate reporting of suspected cases respectively [5]. Many of these instruments were inherited from Nigeria’s impressive response to the Ebola outbreak of 2014, which was described by the WHO as a piece of *‘world-class epidemiological detective work’* [6]. If Ebola, a disease with an average case-fatality rate of 50%, could be defeated within 90 days by Nigeria’s fledgling public health care system [7], certainly COVID-19 which barely kills 5% of infected patients shouldn’t be much of a problem. Well, so it seemed.

## AFTER THE INDEX CASE

Following the arrival of patient zero, the Federal Government instituted the COVID-19 Presidential Task Force (PTF) to provide daily updates on the evolving situation of the outbreak in the country to the public; however, little planning went towards the preparation of palliative measures for the citizens in the event of a nationwide lockdown and enforcement of the self-isolation directive for individuals coming into the country from COVID-19 hotspots was lax. This was evident in the fact that the index case had traveled inter-state on arrival into the country, making primary and secondary contacts and not self-isolating until he became symptomatic. A more confusing scenario was the refusal of the government to institute a travel ban on flights from the most hard-hit countries, until about 3 weeks from the date the first case was reported, a time by which many believed was a little too late [8], as the virus had gained sufficient entry into the country to trigger the ensuing community spread.

## ON TESTING

Prior to the onset of the COVID-19 outbreak the NCDC managed a molecular RT-PCR laboratory network of 6 laboratories in 3 states (out of 36) and the Federal Capital Territory (FCT). Though small, this was sufficient to handle the nation’s diagnostic needs at the time, which mainly involved the diagnosis of Lassa fever, yellow fever and cholera, the 3 major outbreaks the country dealt with periodically. Fortunately, all six laboratories were biosafety level-3 (BSL-3) certified, a step higher than the CDC recommended BSL-2, for routine COVID-19 diagnostic procedures [9]. This meant that these facilities could be deployed immediately for diagnosing COVID-19 cases across the country. They however soon became overwhelmed, leading the NCDC to activate the laboratories of three federal teaching hospitals to bring the total number of COVID-19 diagnostic laboratories to 9 in 6 states, by April, providing a total of 2500 tests per day [10]. As evidence of community transmission emerged, the need to expand testing capacity further in order to gain an accurate picture of case incidence figures became imperative, leading the NCDC to publish a national strategy for the expansion of COVID-19 testing capacity [10]. The plan was simple and it was to activate more molecular RT-PCR laboratories, while leveraging the high-throughput HIV molecular testing laboratories and to repurpose point-of-care tuberculosis GeneXpert testing machines for COVID-19 testing [10]. A critical component needed for the success of this plan however was private sector support; and when the government called, the private sector responded; with both for-profit companies and non-profit organizations such as: Access Bank, 54Gene, Aliko Dangote Foundation and Flying Doctors Ltd, supporting the activation of several mobile diagnostic laboratories in many states across the country [11]. As of August 10, 2020, there were 62 COVID-19 testing facilities in the NCDC network [12].

**Figure:**
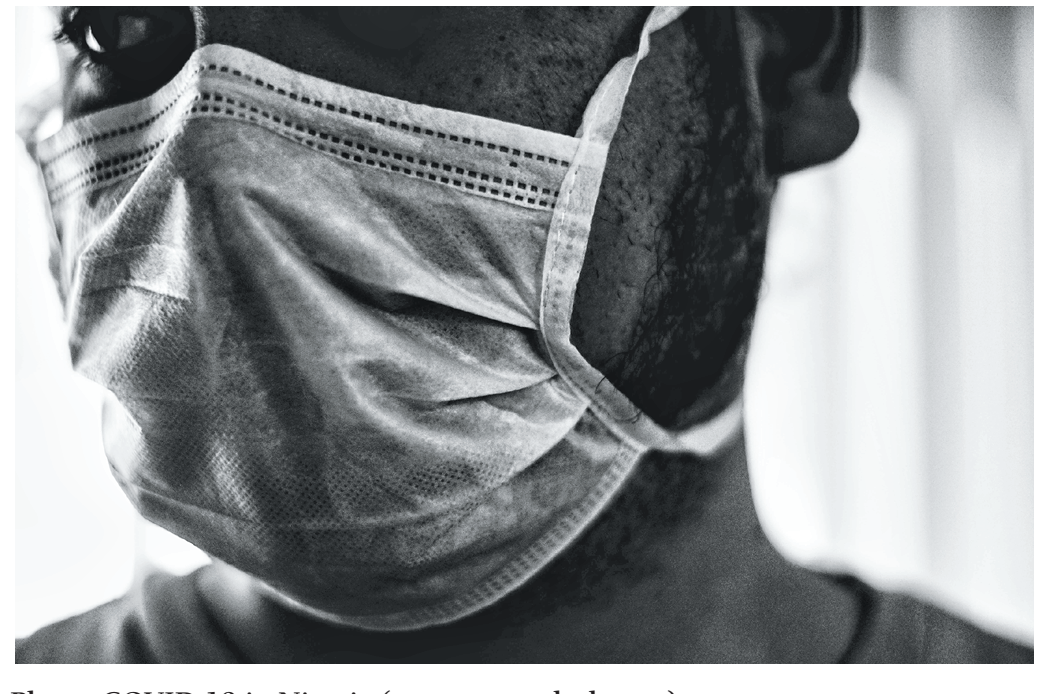
Photo: COVID-19 in Nigeria (source: unsplash.com).

## ON HEALTHCARE SYSTEM PREPAREDNESS

Before the onset of COVID-19, the Nigeria health care system has been plagued by numerous challenges including: shortage of qualified health care personnel, inadequate budgetary allocation to health, dilapidated health care infrastructure and medical tourism (with several Nigerians, especially of the political class preferring to travel overseas for treatment, while leaving the Nigeria health sector in a state of dilapidation). It was expected that a COVID-19 outbreak scenario would be the metaphorical *‘final straw that breaks the camel’s back’*. A recently published nationwide study by Umar et al. [13] investigating the level of preparedness of Nigerian health care facilities towards managing the COVID-19 pandemic highlighted several disappointing but unsurprising findings. Of the 451 health care workers who participated in this survey: only 4.5% were provided with facemasks by their health institutions, while 22.4% had an adequate supply of hand gloves and sanitizers; availability of other personal protective equipment (PPE) was reported by 13.3%, while the accessibility of same was reported by only 6.9% [13]. Only 45.5% confirmed that their facilities had dedicated isolation wards for suspected or confirmed COVID-19 cases, while 20% asserted to the availability of functional intensive care units for COVID-19 patients in critical conditions [13].

## ON SOCIO-ECONOMIC IMPACT AND PALLIATIVE MEASURES

Following an accelerated spread of COVID-19 in Nigeria, the Federal Government ordered an initial 2-week lockdown on March 30th, in 2 states (Lagos and Ogun) as well as the FCT, which was extended by an additional 2 weeks on April 13th. With a teeming population of over 200 million people, 50% of which were living below the international poverty line of US$ 1.25/d [14], the informal economy represents a crucial lifeline for the increasing number of people unable to secure white collar jobs in Nigeria. The National Bureau of Statistics (NBS) reported that the informal sector was responsible for 80% of job creation in 2019 and contributed 58% to the nation’s Gross Domestic Product (GDP) [14]. In lieu of this numbers, the preventive measures associated with the imposed lockdown, such as: movement restrictions, interstate travel ban and physical distancing meant that millions of people in the informal sector, relying on daily income for survival, were rendered unemployed and without a source of income [14]. In recognition of this dilemma, the government had promised palliative measures in form of conditional cash transfers for about 3.6 million vulnerable citizens; however, the eligibility criteria for this measure, including: referral by community leaders and available bank balance below N5000 (US$ 13), as well as unproven claims of misappropriation, made this scheme largely ineffective [15]. This, coupled with human rights violations by security personnel responsible for enforcing the lockdown directive resulted in demonstrations and widespread disobedience of the directive by many, effectively rendering it redundant, and contributing to community transmission of COVID-19 [14].

## CONCLUSION

The summary of evidence suggests that although government preparedness and response efforts to this pandemic have not been outstanding, they have certainly been instrumental towards the recent flattening of the epidemic curve. However, one cannot but wonder how many lives could have been saved, should some of the mistakes highlighted have not been made.
